# Engineering Supramolecular Hybrid Architectures with Directional Organofluorine Bonds

**DOI:** 10.1002/smsc.202300110

**Published:** 2023-12-13

**Authors:** Patience A. Kotei, Daniel W. Paley, Vanessa Oklejas, David W. Mittan-Moreau, Elyse A. Schriber, Mariya Aleksich, Maggie C. Willson, Ichiro Inoue, Shigeki Owada, Kensuke Tono, Michihiro Sugahara, Satomi Inaba-Inoue, Andrew Aquila, Frédéric Poitevin, Johannes P. Blaschke, Stella Lisova, Mark S. Hunter, Raymond G. Sierra, José A. Gascón, Nicholas K. Sauter, Aaron S. Brewster, James Nathan Hohman

**Affiliations:** ^1^ Institute of Materials Science University of Connecticut Storrs CT 06269 USA; ^2^ Department of Chemistry University of Connecticut Storrs CT 06269 USA; ^3^ Molecular Biophysics and Integrated Bioimaging Division Lawrence Berkeley National Laboratory Berkeley CA 94720 USA; ^4^ Advanced Photon Technology Division RIKEN SPring-8 Center 1-1-1 Kouto Sayo Hyogo 679-5148 Japan; ^5^ XFEL Utilization Division Japan Synchrotron Radiation Research Institute 1-1-1 Kouto Sayo Hyogo 679-5198 Japan; ^6^ Structural Biology Research Center Photon Factory Institute of Materials Structure Science High Energy Accelerator Research Organization 1-1 Oho Tsukuba Ibaraki 305-0801 Japan; ^7^ Linac Coherent Light Source SLAC National Accelerator Laboratory Menlo Park CA 94025 USA; ^8^ National Energy Research Scientific Computing Center Lawrence Berkeley National Laboratory Berkeley CA 94720 USA

**Keywords:** C—F bonding, crystal engineering, metal-organic chalcogenolates, small-molecule serial femtosecond crystallography, supramolecular synthons

## Abstract

Understanding how chemical modifications alter the atomic‐scale organization of materials is of fundamental importance in materials engineering and the target of considerable efforts in computational prediction. Incorporating covalent and noncovalent interactions in designing crystals while “piggybacking” on the driving force of molecular self‐assembly has augmented efforts to understand the emergence of complex structures using directed synthesis. In this work, microcrystalline powders of the silver 2‐, 3‐, and 4‐fluorobenzenethiolates are prepared and their structures are resolved by small‐molecule serial femtosecond X‐ray crystallography. These three compounds enable the emergence and role of supramolecular synthons in the crystal structures of 3D metal‐organic chalcogenolates to be examined. The unique divergence in their optoelectronic, morphological, and structural behaviors is assessed. The extent of C—H—F interactions and their influence on the structure and the observed trends in the thermal stability of the crystals are quantified through theoretical calculations and thermogravimetric analysis.

## Graphical abstract

Exploiting noncovalent interactions in designing functional hybrid material systems is an attractive and efficient process for gaining atomic‐level organization. Modifying the nature, steric factors, and electronegativity of functional groups used in building supramolecular architectures serves as a frontier to understanding material properties and how to arrange them into remarkable functional materials.

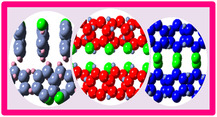

## 1 Introduction

Synthons are highly conserved supramolecular patterns that emerge in crystal structures of related molecules.^[^ ^1^, ^2^ ^]^ Synthons enable the classification of recurring patterns for crystal engineering because they give a systematic approach to understanding how different molecules self‐organize.^[^ ^3^, ^4^ ^]^ The rise of hybrid architectures such as metal–organic chalcogenolates (MOChas) that yield well‐defined and highly inter‐related supramolecular and coordinated phases present an opportunity to examine the roles played by synthons in the emergence of inorganic structure in complex materials. However, it has been challenging to relate compatible synthons to inorganic structures because the small crystal sizes endemic to MOChas make structural solutions challenging to acquire. We have recently demonstrated that small‐molecule serial femtosecond crystallography (smSFX) is useful for accelerating the characterization of compounds in this material class.^[^ ^5^ ^]^ Here, we use this approach to perform microcrystallography on the three silver fluorobenzenethiolates. Each isomer yields distinct crystal Bravais lattice, spacegroup, and habit. The detailed structures reveal how the patterns of CF—H hydrogen bonding relate to the inorganic nanostructures in the context of the alignments and energies of the interactions of the supramolecular phase. These structures reveal C—H–F hydrogen bonding and its role in modulating the inorganic structures. The interaction strengths are evaluated in the context of material thermal stability. Because fluorine is isosteric with hydrogen,^[^ ^6^ ^]^ we can examine the role of the dipolar interactions largely in isolation of steric factors in MOChas.^[^ ^7^, ^8^ ^]^ We identify compatibilities between organic synthons and the resulting inorganic architectures and conclude with a discussion of the supramolecular synthons that coexist with the low‐dimensional inorganic structure. This work provides a foundation for a systematic approach to the reticular design of hybrid materials with both supramolecular and inorganic components.

The MOChas are self‐assembling hybrid materials that form 0D complexes and clusters, 1D polymers, and 2D sheets depending on the steric demands of the organic ligands.^[^ ^8^ ^]^ These compounds have recently attracted interest for excitonic photophysical properties expressed as their bulk 3D crystals.^[^ ^9^, ^10^, ^11^, ^12^ ^]^ This unique arrangement of monolayer performance in 3D crystals and numerous opportunities for crystal engineering by metal,^[^ ^13^ ^]^ chalcogen,^[^ ^14^, ^15^ ^]^ and ligand (organic group) substitution makes the system an ideal test platform for understanding the emergence of complex/function properties in hybrid quantum solids.^[^ ^16^, ^17^ ^]^

The C—F bond has previously been used as a noncovalent interaction for materials engineering,^[^ ^18^ ^]^ and the complex behavior of dipolar–dipolar interactions realized in crystal systems has been recognized.^[^ ^19^, ^20^ ^]^ Bond–dipole interactions have been observed to be more complicated^[^ ^21^, ^22^ ^]^ than molecular dipolar interactions, which generally do not play a pivotal role in crystal packing.^[^ ^23^, ^24^ ^]^ Conversely, both hydrogen and halogen bonding are reliable tools in crystal engineering for directing supramolecular self‐assembly.^[^ ^25^, ^26^, ^27^ ^]^ The variation in their steric bulk and electronegativity makes them attractive candidates in the manipulation of crystal packing and geometry.^[^ ^28^, ^29^ ^]^ C—F bond functionalization has also been extensively used in the modification of materials to improve optoelectronics, structural stability, surface activity, and processability in layered materials such as graphene and its derivatives,^[^ ^30^, ^31^ ^]^ 2D‐perovskites,^[^ ^32^, ^33^ ^]^ transition metal dichalcogenides,^[^ ^34^, ^35^ ^]^ transition metal oxides,^[^ ^36^, ^37^ ^]^ metal–organic frameworks, and porous coordination polymers.^[^ ^38^, ^39^ ^]^

## 2 Results and Discussion

### 2.1 Synthesis and Purification of Fluorinated Silver Benzenethiolates

**Figure 1:**
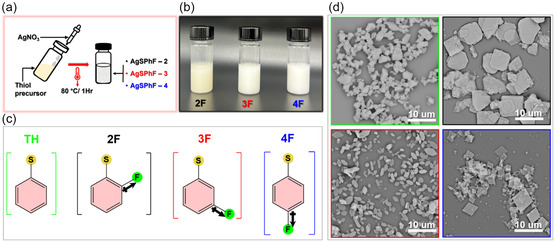
Synthesis. a) Schematic representation of the synthetic method used, b) cream–to‐white color of fluorinated silver benzenethiolate crystals in methanol, c) thiol precursors showing the different C—F bond dipole direction in the 2, 3, and 4 positions, and d) morphological variation in TH, 2F, 3F, and 4F (neon green color represents TH, black 2F, red 3F, and blue 4F).

An overview of the synthetic scheme, precursors, and products is shown in **Figure** **1**. For this work, we prepared silver 2‐fluorobenzenethiolate, silver 3‐fluorobenzenethiolate, and silver 4‐fluorobenzenetholate; hereafter referred to as 2F, 3F, and 4F, respectively. We use silver benzenethiolate (thiorene (TH)) as the nonfunctional control example. White crystalline powders of these 2D layered compounds were recovered after the reaction of silver nitrate with the corresponding ligand in acetonitrile. Suspensions of microcrystals used for crystallographic analysis by smSFX are shown in Figure 1b. Relevant bond dipole orientations of the ligands with respect to the sulfur atom are depicted in Figure 1c.

Representative scanning electron micrographs of the products 2F, 3F, and 4F and their typical crystal habits are also shown in Figure 1d. Note the change in crystal habit from tabular in 2F, pointed‐rectangular in 3F, and back to tabular in 4F. The peculiar oval‐like habit of the 3F crystals shown suggests the trend toward elongation, with the curved edges implying a fast growth direction.

### 2.2 Ensemble Measurements of the Ligand‐Specific Phenomena

**Figure 2:**
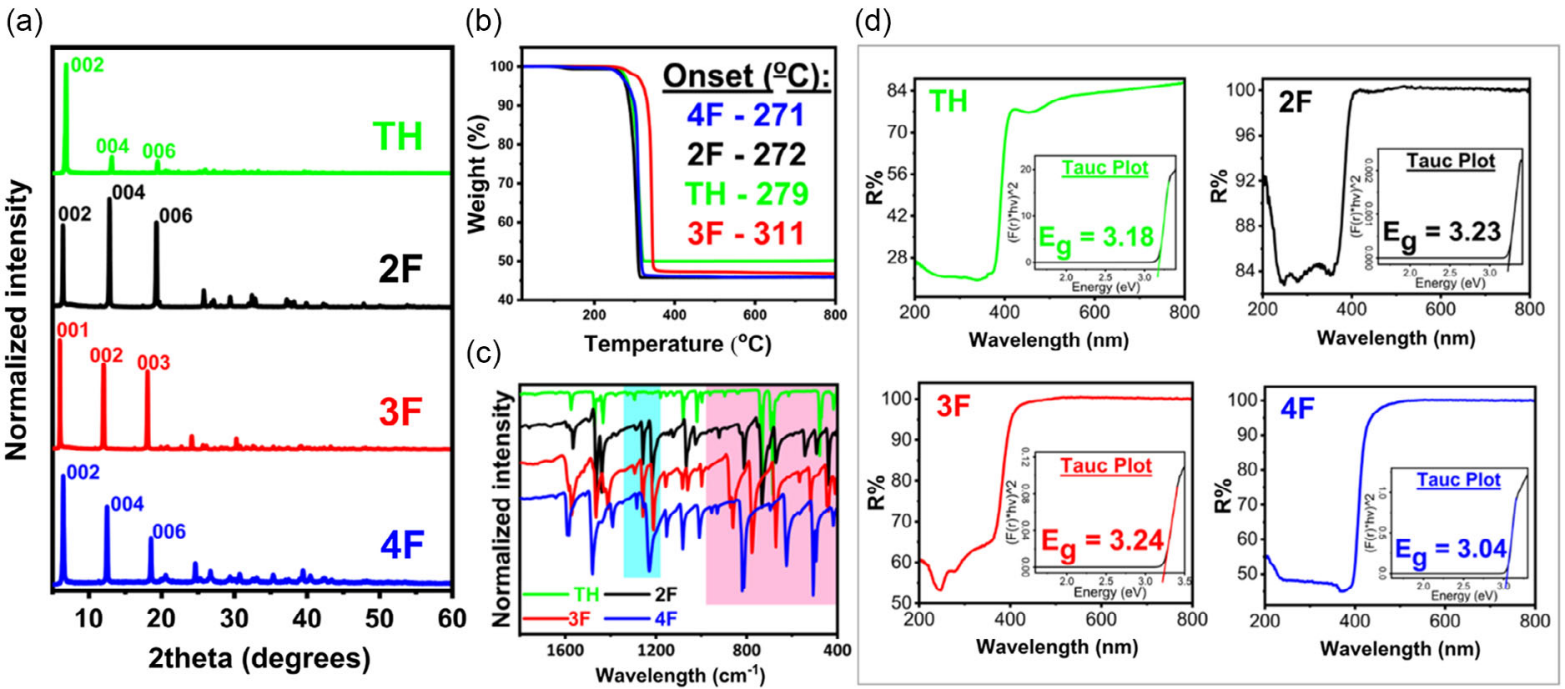
Secondary characterization. a) The three evenly spaced powder XRD reflections present in all four samples are indicative of layered solids. Note the index labels are different in the 3F case because of their unique unit cell and space group. b) 3F is distinguished in thermogravimetric analysis by a higher decomposition onset temperature. c) IR spectra of samples. The cyan region is attributed to C—H···F contacts, the pink region shows the distinct fingerprints of each structure. d) Reflectance spectra and optical bandgaps of TH, 2F, 3F, and 4F.

Microcrystals were recovered in the 3–10 μm range and in high purity. The finely powdered products were used directly for further characterization by UV–vis, pXRD, Fourier transformed infrared (FTIR) spectroscopy, and thermogravimetric analysis (TGA), and these results are collected in **Figure** **2**. The regular spacing of low‐angle peaks in the pXRD patterns (Figure 2a) is characteristic of stacked/layered materials.^[^ ^9^ ^]^

TGA experiments result in complete decomposition and loss of the organic and sulfur components, leaving a residual attributed to the silver metal with a value close to that expected from stoichiometry (Table S1, Supporting Information). The 2F, 4F, and the TH control samples all share a similar decomposition temperature at 180 °C (Table S1, Supporting Information). The 3F is an outlier with a higher thermal decomposition temperature of 210 °C. Because of the similarity in composition, this difference may be due to an enhancement of stability in the 3F system and might be attributable to the stabilization of either the metal–sulfur bonding or to the supramolecular lattice. To explain this discrepancy, we turn to the acquisition of crystal structural data and theoretical calculations.

**Table 1:** Data collection and refinement statistics

Compound	2F	3F	4F
Formula	AgSC_6_H_4_F	AgSC_6_H_4_F	AgSC_6_H_4_F
MW	235.02	235.02	235.02
Space group	$P\bar{4}2_{1}c$	*P*2_1_	*Cmce*
*a* [Å]	6.717	7.371	6.023
*b* [Å]	6.717	5.935	7.262
*c* [Å]	27.443	14.887	29.520
*α* [°]	90	90	90
*β* [°]	90	95.45	90
*γ* [°]	90	90	90
*V* [Å^3^]	1238.1	648.3	1291.2
*Z*	8	4	8
*ρ*_calc_ [g cm^−3^]	2.522	2.408	2.418
XFEL source	SACLA	LCLS	LCLS
*λ* [Å]	0.79887	0.69465	0.69458
*T* [K]	298	298	298
*d*_min_ [Å]	0.83	0.83	0.83
*μ* [mm^1^]	4.845	3.159	3.168
Frames	520 000	308 401	980 726
Crystals	2644	6283	6414
Time [min]	289	43	136
Data	679	1305	657
Restraints	1	91	46
Parameters	37	163	64
*R*_1_(obs) [%]	14.6	9.05	9.63
*R*_1_(all) [%]	22.6	9.78	13.3
*S*	1.46	1.13	1.11
Peak, hole [e^−^ Å^−3^]	1.58, −1.44	1.81, −1.03	.72, ‐.60

The vibrational spectra for each compound show a fingerprint with strong absorptions for the TH, 2F, 3F, and 4F structures. Bands highlighted in pink in Figure 2c (400–1000 cm^−1^) show notable differences in the vibrational modes. C—F symmetric and asymmetric stretching modes, *v*(C—F) 1000–1400 cm^−1^, are assigned (observed in the unhighlighted region) after inferring from the literature.^[^ ^40^, ^41^ ^]^ Ag—S bonds are assigned (within the pink highlighted region) to the stretches falling below 500 cm^−1^, with a variation in position observed across the series. The band absorption edge for all compounds falls between 3.0 and 3.2 eV (Figure 2d), consistent with white, crystalline solids. The intricate details about how the crystal structures obtained correspond to their infrared (IR) spectra and TGA data will be highlighted further in this report.

### 2.3 Structure Determination of 2F, 3F, and 4F

The smSFX technique used was described by Schriber et al.^[^ ^5^ ^]^ Experimental metrology was refined by the process used by Brewster et al.^[^ ^42^ ^]^ *Dials.stills_process* ^[^ ^43^, ^44^ ^]^ was used for spotfinding and indexing via the *cctbx.xfel.small_cell_process* wrapper that adds maximum clique indexing for sparse patterns.^[^ ^45^ ^]^ TOPAS‐Academic^[^ ^46^ ^]^ was used to determine unit cells. *Cctbx.xfel.merge* was used for scaling and merging. ShelXL,^[^ ^47^ ^]^ ShelXT,^[^ ^47^ ^]^ Superflip,^[^ ^48^ ^]^ Olex2,^[^ ^49^ ^]^ and Platon^[^ ^50^ ^]^ were used for structure solution and refinement. The data processing procedure follows steps that have been previously described^[^ ^51^ ^]^ and are reproduced in the Supporting Information. Data collection and refinement statistics are collected in **Table** **1**.

3F and 4F were solved and refined by generally standard techniques. The structure solution was in ShelXT with the default parameters. For 4F, the fluorophenyl group was disordered by symmetry and it was necessary to constrain the C‐atom positions in a regular hexagon (AFIX 66). All nonhydrogen atomic displacement parameters were refined anisotropically with rigid‐bond restraints (RIGU) on the carbon and fluorine atoms. Hydrogen atoms were placed in calculated positions and refined with riding coordinates and atomic displacement parameters (ADPs).

2F failed to solve in ShelXT in many repeated trials with various parameters. Our solution was obtained by running repeated trials in Superflip. For each candidate, all atoms except silver were deleted and a refinement was attempted. The solutions were evaluated for refinement stability, map quality, and chemical reasonableness. The most reasonable solution was obtained in $P\bar{4}2_{1}c$ with two independent silver positions and one arylthiol group in the asymmetric unit. The refinement was completed with anisotropic ADPs for the heavy atoms and isotropic ADPs for carbon and fluorine. The aryl ring was constrained to a regular hexagon. Hydrogen atoms were refined with a riding model as above. We note that this structure determination for 2F was particularly challenging, and we consider it possible that the space group is incorrect. The agreement factors (*R*_1_ ≈ 15%) are only slightly elevated in comparison to other structures from the same experiment, but the refinement was somewhat unstable and the atomic displacement parameters show some signs of pathology. In contrast, we also note that in the Cambridge structural database our proposed space group, $P\bar{4}2_{1}c$, is the sixth most common tetragonal space group (out of 68) and the 32^nd^ most common overall. One plausible guess is that the true symmetry is in Laue group 4 m^−1^ with pseudosymmetry that approximates the extra elements .2_1_. and .*c* given here. In this scenario, our dataset would appear perfectly twinned due to the unknown orientation of individual still shots about the possible “twin elements”, e.g., 2[011]. This “twinning” could explain our failure to obtain a solution in the lower Laue group, and the incorrect symmetry would explain the unstable ADPs and elevated *R*‐factors.

Overall, we have high confidence in our proposed structure of 2F as a schematic depiction (sometimes called a “connectivity‐quality structure”), but all details should be treated with caution. We report silver–silver distances with two decimal places, but it is probably impossible to meaningfully quantify the uncertainties on the measurements.

### 2.4 Observed Silver–Silver Contacts

Argentophilic Ag···Ag contacts shorter than 3.5 Å have been identified in a variety of luminescent coordination complexes^[^ ^52^ ^]^ and in mithrene.^[^ ^12^ ^]^ Argentophilic interactions are increasingly observed in low‐dimensional materials and may play a vital role in the stabilization of bonding networks.^[^ ^53^ ^]^ The coordinative flexibility of silver atoms contributes to geometrical diversity, with a rich variety of interactions between ligands and metal centers.^[^ ^54^ ^]^

**Figure 3:**
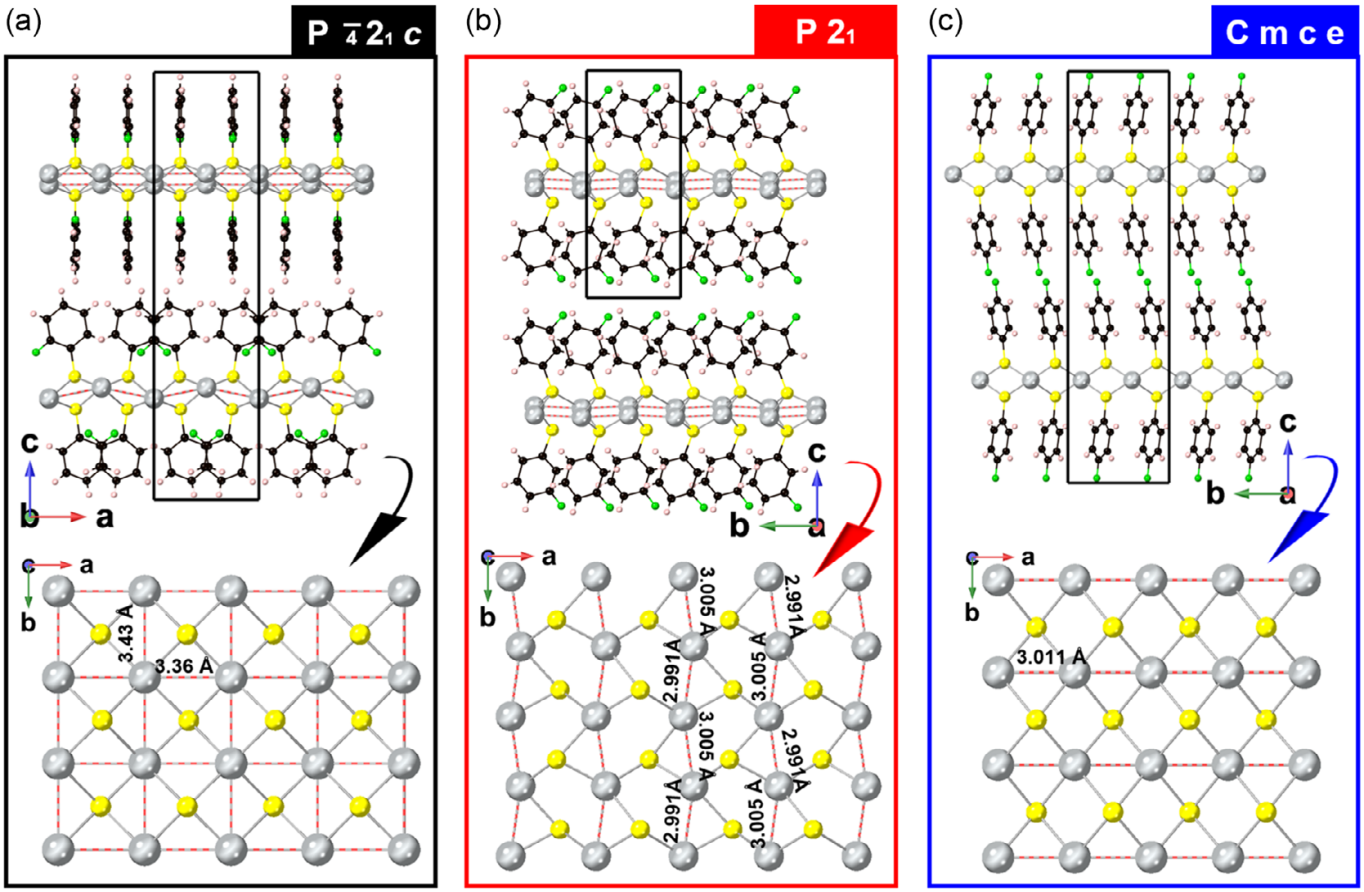
Crystal structures of 2F, 3F, and 4F. Unit cells are shown in black box. a) Structure of 2F looking down the b‐plane, b) structure of 3F looking down the a‐plane, and c) structure of 4F looking down the b‐plane. A spread of possible silver–silver interactions present in 2F, 3F, and 4F is shown beneath each respective crystal structure. Note the shorter structures are shown without organic components for clarity. The Ag···Ag network in the 3F system appears to be folded. This distortion (with respect to the linear arrangement observed in 2F and 4F) may have been realized to satisfy chemical and geometric demands imposed on the supramolecular structure.

Argentophilic interactions are sensitive to their environments, hence, the packing of ligands in a system can influence the metallophilic contacts.^[^ ^55^ ^]^ We observe the modification of this contact environment by comparing the three fluorine‐substituted positional isomers. The overall crystal structures of the three materials are shown in **Figure** **3**. Yunus et al., through calculations at the density functional theory (DFT) level, showed the shortest Ag···Ag interactions between 2.91 and 2.98 Å have the most stabilization energy value.^[^ ^53^ ^]^ Meaningful contacts such as these have significant directional and stabilizing effects on the crystal matrix. Thus, meaningful argentophilic contacts are not observed in 2F, possibly because of the closeness of the C—F bond dipole (packing forces) to the silver metal being disruptive. The lower bond length values observed in 3F fall within the range considered to be significant. In the absence of any meaningful interactions, they may contribute to the stability of the 2D networks and 3D supramolecular frameworks. This observation agrees with the observed increased thermal stability of 3F.

### 2.5 Observed C–F···H Interactions in 2F, 3F, and 4F Crystal Structures

While C—H–F hydrogen bonding is weak, this interaction can guide molecular association and alter molecular and supramolecular architectures.^[^ ^56^ ^]^ These interactions have modest energy values ranging between of 2 and 20 kJ mol^−1^, yet their effect on crystal packing is equally relevant to more typical hydrogen bonds in other crystal systems.^[^ ^57^, ^58^ ^]^ Here, we consider the role of the fluorine position on the energy and patterns formed by C—H–F contacts. These contacts and patterns may evolve because of how the crystals pack/ assemble in the supramolecular architecture (not necessarily H‐bonding).

A summary of C—H–F bond distances (intra‐ and intermolecular) and angles in the Cambridge Structural Database (CSD) has been compiled by Hulliger et al.^[^ ^59^ ^]^ The C—H–F bond distance in 2F is 2.680 Å, while short and long intermolecular contacts [2.434 and 2.841] Å are observed in 3F. 4F also exhibits both short and long C—H–F contacts [2.658 and 2.892] Å. These contacts observed all fall within the range reported. This may not have any substantial effect on the crystal structure because they may be weak in nature due to the poor electron‐donating property of fluorine (F) bonded to C (sp^2^). This reluctance may result from the lone pairs on fluorine conjugating with the aromatic pi‐electron cloud, fully utilizing its negative inductive and positive mesomeric potentials.

**Figure 4:**
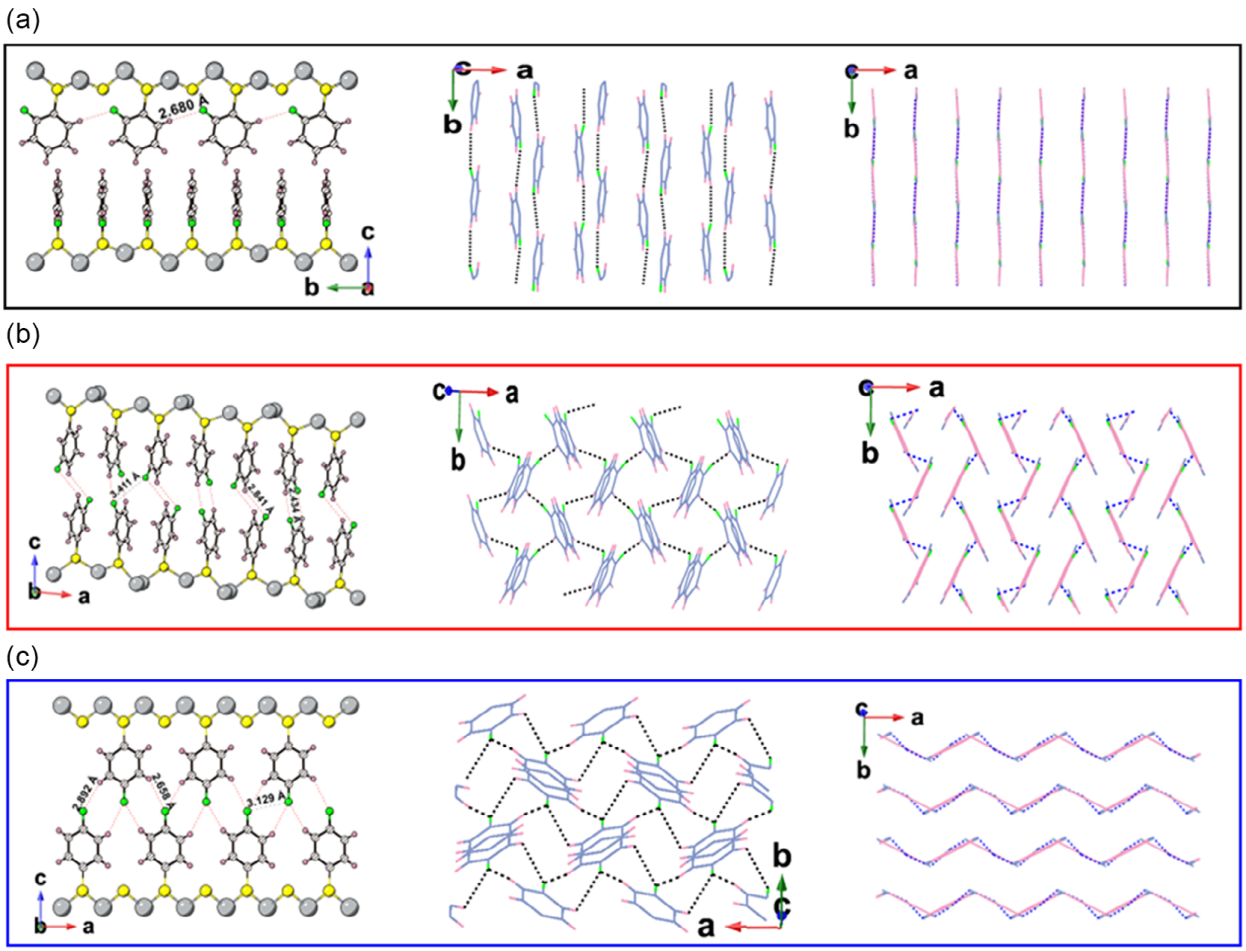
C—H···F synthons. a) 2F intermolecular C—H···F contacts (2.680 Å). A linear supramolecular synthon is observed in 2F. b) Short and long intermolecular contacts [2.434 and 2.841] Å observed in 3F. A type II supramolecular synthon (catemeric) with a sandwich‐herringbone arrangement of organic groups is seen. c) 4F exhibits both short and long C—H···F contacts [2.658 and 2.892] Å. The fluorine environment is bifurcated. A type I supramolecular synthon (catemeric) and a herringbone arrangement of organic groups are observed.

Desiraju et al. have investigated C—H–F contacts in several fluorobenzene analogs with some showing bifurcations using IR spectroscopy.^[^ ^60^ ^]^ They have also shown that C‐F groups will prefer C—F···H interactions (hydrogen bonding) rather than F–F interactions (halogen bonding). This makes the behavior of fluorine in crystal packing different from its heavier halogen counterparts. Thus, the F–F contacts (**Figure** **4**) observed in 3F (3.411 Å) and in 4F (3.129 Å), although they fall within the range for fluorine–halogen bonding, they may have resulted from crystal packing considerations, with no significant stabilization effect. Using FTIR analysis, as shown in Figure 2c (blue highlighted region), we identified C–H–F contacts between 1200 and 1300 cm^−1^. As shown in **Table** **2**, bifurcations draw out more electrons from the contributing atoms, leading to weaker bonds and lower bond vibrations. With the observed C—H–F contacts in the 4F system and the bifurcated fluorine environment, this observation agrees with the lower bond vibrations observed in its IR spectrum.

**Table 2:** Possible C—H···F contacts confirmed with IR data. A bifurcated fluorine environment is observed in the 4‐fluoro analog

IR characteristics of samples			
Sample	C—H environment	F environment	C‐F…H (cm^−1^)
2F	Nonbifurcated	Nonbifurcated	1290, 1260, 1218
3F	Nonbifurcated	Nonbifurcated	1295, 1260, 1214
4F	Nonbifurcated	Bifurcated	1284, 1259, 1231

### 2.6 Aromatic Packing Motifs in 2F, 3F, and 4F Crystal Structures

Crystal structures usually pack as close as possible, with deviations being attributed to diversity in the chemical makeup of crystals. Highly directional atom‐specific (bonding) interactions are strong and kinetically favorable. With the incorporation of fluorine (isosteric with hydrogen) and its positioning in these supramolecules, alternative structures with different densities and packing/stacking may be realized. As hybrid structures, knowledge of how the organic groups pack is of vital interest because intermolecular interactions between crystal packing motifs can lead to the emergence of other energetically favored configurations (polymorphism). Without this information, the understanding, design, and structure prediction of this material class can be rendered challenging and tedious.

In a previous study, Hohman et al. demonstrated the origin of the optoelectronic property observed in the archetype (silver benzeneselenolate), thus, attributing the new properties exhibited by members in this series to the inorganic framework was obvious from the outset.^[^ ^5^ ^]^ As a result of the elusive nature of typical C–H–F interactions, studies done by Desiraju et al. in evaluating the role of C–H–F interactions in the crystal structures were key in our reconciliation of crystal structures and their thermal stabilities.^[^ ^61^ ^]^ Additionally, Desiraju and Gavezotti drew empirical correlations between molecules and the resulting packing motifs. The common crystal packing motifs they identified now serve as a benchmark in analyzing the relationship between structure and packing.^[^ ^62^ ^]^

As they observed in several fluorobenzenes, we observed similar C–H–F supramolecular synthons in our three analogs (Figure 4). A linear synthon is observed in 2F, coupled with the unique 180° rotation of subsequent layers, and a mesh‐like arrangement of the aromatic groups present is observed. We identified their Type II synthon in the 3F analogue leading to a sandwich‐herringbone arrangement of the aromatic groups. The enhanced thermal stability of 3F could also be attributed to the presence of the short C–H–F (shortest observed in the entire series) interactions and the packing motif realized in its architecture. Although the fluorine environment is bifurcated in the 4F analog, we observed their Type I synthon which was relatively weak interactions with the herringbone arrangement of the aromatic group dominating. Among the members in the series, the shortest F–F contact is observed in 4F. Studies have established that fluorine is not likely to form F–F halogen bonds because it is not easily polarizable, thus, this short contact may be due to geometric considerations (crystal packing forces).

### 2.7 DFT Calculations

The TGA analysis reveals that the 3F crystal isomer exhibits superior thermal stability compared to the 2F and 4F counterparts. To investigate this further, we utilized a plane‐wave pseudopotential formalism to calculate the energy per unit cell for all three crystals. During the computation, we relaxed all atoms and unit cell parameters while preserving the original crystal symmetry. The resulting data, presented in Table S2, Supporting Information, include the key distances that characterize the supramolecular synthons. The computed distances align with the crystallographic data and the variability in the C—H–F patterns. Specifically, the 3F system shows the shortest C—H–F distance (2.25 Å), confirming the stabilizing role of C—H–F bonds. While the DFT C—H–F distances are underestimated by approximately 7%, this is, in part, the result of an underestimation of the C—H bond lengths in the crystal structure. It is known that C—H bonds are typically underestimated by ≈0.1 Å. The energy per unit cell per atom is lowest for the 3F system, followed by 4F and 2F, consistent with the TGA analysis.

### 2.8 Luminescent Polymorph of the 2F System

**Figure 5:**
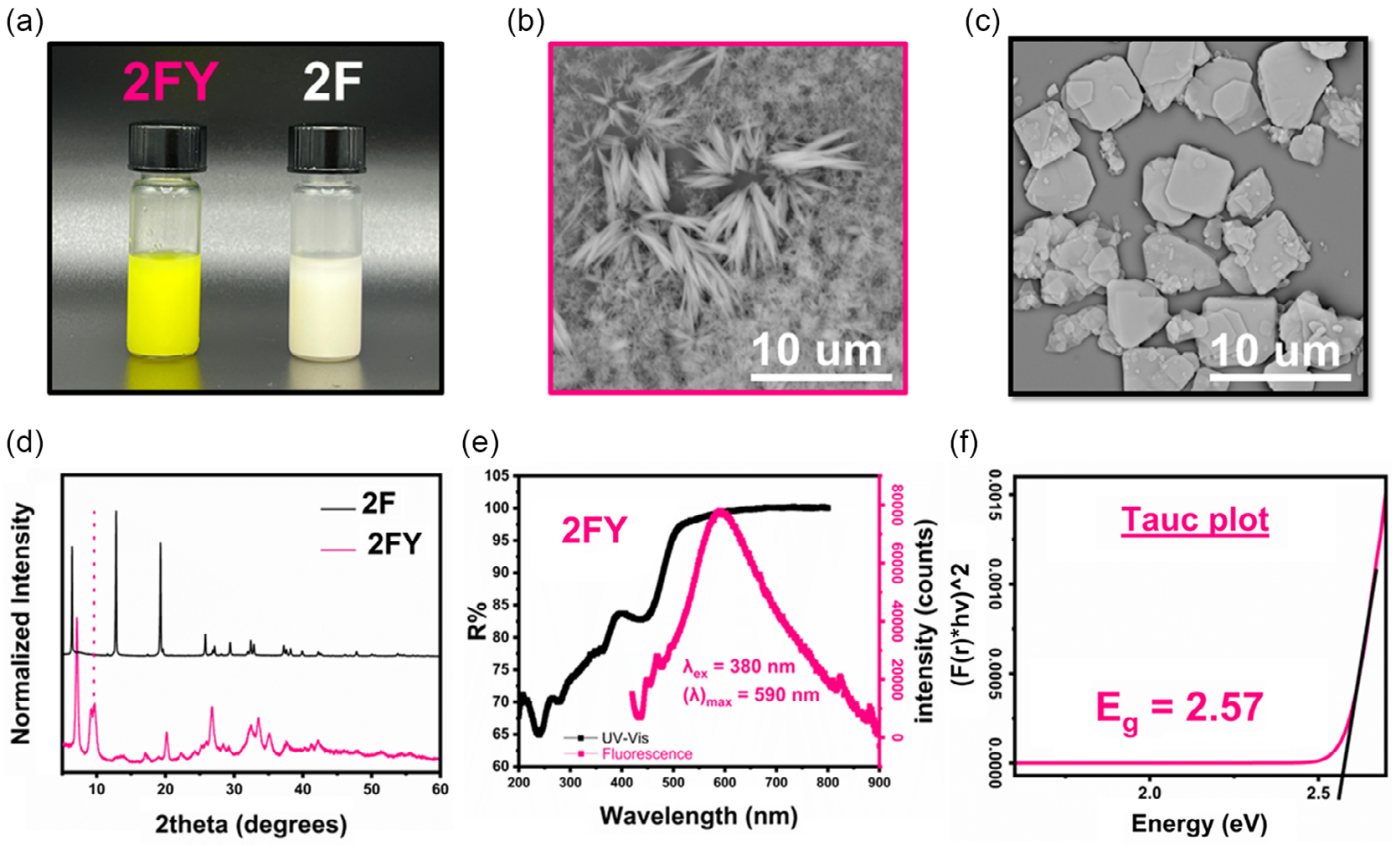
a) Variation in 2‐fluoro color change upon sonicating or heating the 2F after synthesis. b,c) Morphological change from 2FY to 2F. d) Difference in the diffraction pattern of the 2FY and 2F polymorphs. e) Luminescent property of 2FY divergent from 2F. f) The yellow analog (2FY) possesses a smaller bandgap.

A polymorphic behavior is observed in the 2F case, with a second, intensely yellow product (2FY) appearing under certain conditions (**Figure** **5**a), and this needed to first be removed. Unlike the other compounds, the powder diffraction of crude 2FY product reveals additional reflections consistent with a mixture of phases. The 2FY product is unstable to both heating and to sonication. Both postsynthetic treatments (sonication or heating) yield phase‐pure 2F, yielding the diffraction pattern in Figure 5d. After purification, both the X‐ray and optical signatures of the 2FY are largely absent, and all the crystals uniformly have the tabular habit (Figure 5c). Interestingly, the tabular crystal habit of the treated 2F crystals suggests elongation at the crystal edges relative to the pretreatment crystals. 2FY therefore appears to have been decomposed and added to the 2F phase crystals.

We do not include microcrystallographic analysis of the 2FY in this work as we had not isolated it before smSFX experiments, but in our synthetic follow‐up efforts, we were able to obtain pure samples of 2FY. This enabled comparison of the FTIR spectra of the two phases (2FY and 2F). The spectra of the two phases are nominally indistinguishable, except for a shift in the peaks associated with the Ag‐S phase as shown in Figure S2, Supporting Information. The emergence of a new diffraction peak, a 1D crystal habit, and a new optical phenomena lead us to suggest 2FY is a distinct 1D phase of 2F. This has precedence in the literature for a doubly fluorinated silver 2,6 difluorophenylselenolate,^[^ ^17^ ^]^ so we postulate that a similar phase is accessible for the singly fluorinated species.

## 3 Conclusion

Rational material design is a challenging problem because of the sheer diversity of possible configurations of matter. The ease of functionalization of the organic groups (R) attached to the inorganic layers in MOChas makes those interesting targets for crystal engineering. Introducing new chemical functional groups introduces two changes to the target system: the geometric demands of the group itself and the intermolecular forces it exerts on other neighbors in the crystal. This study demonstrates the potential use of C‐F groups as a design tool that can be finely manipulated in the crystal engineering of hybrid systems. We characterized the energetics in the context of the crystal structures.

## 4 Experimental Section

4.1

4.1.1

##### Chemicals Used

Silver nitrate (AgNO_3_, ≥99.0%), acetonitrile (C_2_H_3_N, 99.9%), acetone (C_3_H_6_O, ≥99.9), and 2‐fluorobenzenethiol (2‐C_6_H_4_FSH, 97%) were purchased from Millipore Sigma. Ethanol (C_2_H_5_OH, 100%), 3‐fluorobenzenethiol (3‐C_6_H_4_FSH, 97.0+%), and 4‐fluorobenzenethiol (4‐C_6_H_4_FSH, 97%) were purchased from Fischer Scientific. All chemicals were used as received.

##### Synthesis/Preparation of C_6_H_4_FSAg

The silver precursor solution was prepared by dissolving silver nitrate (50 mg) in acetonitrile (10 mL) in a 50 mL vial. The thiol solution was prepared by combining 46 uL of X‐fluorobenzenethiol (where *X* = 2,3,4) to 10 mL acetonitrile in a beaker. The silver precursor solution was added to the thiol solution in a dropwise fashion. The resulting mixture was heated for 1 h between 80 and 100 °C (Figure 1a). The silver 3‐ and 4‐fluorobenzenethiolates (3F and 4F) yield a milky‐to‐white solid product (Figure 1b). Silver 2‐fluorobenzenethiolate yields a delicate, bright yellow solid (Figure S1, Supporting Information). The yellow color is attributed to the first product identified by X‐ray diffraction (XRD). The yellow product (2FY) is eliminated on heating or sonication to obtain a white product (2F), as shown in Figure S1, Supporting Information. The signature of the yellow product is eliminated as observed in the diffraction pattern (Figure S1, Supporting Information). Silver benzenethiolate (TH) was synthesized as previously the method reported.^[^ ^5^ ^]^

##### Postsynthesis/Purification of C_6_H_4_FSAg

The precipitate was isolated by filtration. The precipitates were double‐washed with acetone to remove any excess precursor present. Ethanol was added to the solid obtained and sonicated for 30 min. The crystals obtained were separated from the ethanol solution by filtration and air‐dried. The crystals were deposited on silicon wafer chips (5 × 5 mm) before analysis (Ted Pella, CA) by drop casting from alcohol suspension for imaging and analysis.

##### Instrumentation

SEM‐EDX analysis was conducted on a Phenom‐World ProX Tabletop Scanning Electron Microscope. The elemental composition of the crystals was determined and quantified using the Phenom ProSuite software at 15 kV. Diffuse reflectance and absorbance ultraviolet–visible (DR UV–vis) spectra were obtained on a Shimadzu UV‐2450 instrument from 200 to 800 nm. The decomposition pathway and thermal stability of the fluorinated benzenethiolates were ascertained with a TGA‐Q500 Thermobalance from TA instruments. The sample was heated from 25 to 800 °C under a flow of nitrogen gas. Infrared spectra of the fluorinated benzenethiolates were obtained from a Thermo Fisher Nicolet 560 w/Specac Quest ATR Accessory Spectrometer (Diamond ATR). The samples were scanned as solids from 4000 to 400 cm^−1^. The different peaks from the spectra were compared to reported values to identify the different bond vibrations in the samples. pXRD analysis was performed with a Bruker D2 Phaser Diffractometer equipped with a high‐speed linear detector (LYNXEYE) and Cu‐Kα radiation (*λ* = 1.54184 Å) at 30 kV and 10 mA. The samples were carefully packed into MTI Zero Diffraction Plate for XRD: 17.8 Dia x1.0 mm with Cavity 10 IDx0.1 mm Si Crystal for the analysis.

##### Density Functional Theory Calculations

All DFT calculations were performed using the plane‐wave pseudopotential formalism in Quantum Espresso (10.1088/0953‐8984/21/39/395 502), integrated in the Schrodinger Suite 2022‐4. The GGA‐PBE level of theory was used with dispersion corrections (DFT‐D3). Energy cutoffs of 60 and 300 Ry were employed for the wave function and charge density, respectively. The PS Library of Dal Corso^[^ ^63^ ^]^ was used for the application of pseudopotentials.

##### smSFX at SACLA

Data collection was performed at the Experimental Hutch 3/Beamline 2 at SACLA.^[^ ^64^ ^]^ Microcrystals were suspended in compatible carrier solvents and delivered to the X‐ray interaction region by a liquid jet sample delivery system with nozzle apertures of 75, 100, 150, and 200 μm.^[^ ^65^ ^]^ The jet width and stability were controlled via He flow rate that ranged between 0.5 and 0.8 L min^−1^ with a steady pressure of 100 psi for the duration of the experiment. The liquid flow rate ranged between 0.1 and 0.25 mL min^−1^. Suspensions with particle concentrations of 1–4 mg mL^−1^ in 0.2% w/v aqueous surfactant solution were sonicated for 30 min before being aliquoted into the sample reservoir. The 30 Hz repetition rate at SACLA allows for more flexibility with jet velocity, so larger jets could be used without a loss in hit rate. We also note that the nozzle aperture and jet width used at SACLA are well‐suited for samples with larger crystallite size distribution and less well‐characterized samples. Lysozyme microcrystals were prepared using a standard protocol and delivered to the interaction as a calibrant for detector panel metrology.

Single‐shot diffraction patterns were recorded on an octal MPCCD detector^[^ ^66^ ^]^ positioned ≈51 mm from the interaction region with a vertical offset of 40 mm and a horizontal offset of 3 mm using the DAPHNIS platform.^[^ ^67^ ^]^ The photon energy of the X‐ray free electron laser (XFEL) pulses was ≈15.5 keV (0.8051 *Å*) and the beam size at the interaction region was focused to 1.2 μm using Kirkpatrick–Baez focusing mirror optics.^[^ ^68^ ^]^ Each pulse had a duration below 10 fs^[^ ^69^, ^70^ ^]^ with a per‐pulse energy of 0.3 m**J**. The XFEL photon flux (10^11^ photons/pulse) was not attenuated. All data collection was performed in atmospheric conditions and under room temperature.

##### smSFX at Linac Coherent Light Source (LCLS)

Data collection was performed at the coherent X‐ray imaging (CXI) endstation at the LCLS.^[^ ^71^ ^]^ Microcrystals were suspended in compatible carrier solvents and delivered to the XFEL interaction region by a gas dynamic virtual nozzle (GDVN) liquid jet sample delivery system.^[^ ^72^ ^]^ Aqueous surfactant solution (0.2–0.3% w/v) and water were used as carrier solvents, determined by their suspending power and compatibility with GDVN nozzles. The focusing He gas pressure varied from 300 to 400 psi and the liquid flow rate was between 30 and 50 μL min^−1^. Sample concentration varied between 3 and 4 mg mL^−1^ depending on particle behavior in the suspension. Thaumatin microcrystals were prepared using a standard protocol^[^ ^73^ ^]^ and delivered to the interaction region as a calibrant for detector panel metrology at LY65.

Single‐shot diffraction patterns were recorded on a Jungfrau 4M detector^[^ ^74^ ^]^ positioned ≈59 and ≈160 mm from the interaction region to record low‐resolution reflections. The photon energy of the XFEL pulses was ≈18 keV (0.686 Å). The beam size at the interaction region was ≈1 μm using beryllium lens focusing optics. The pulse power and photon energy were characterized by a downstream single‐shot spectrometer.^[^ ^75^ ^]^ The repetition rate of the XFEL was 120 Hz, delivering 120 pulses per second with a pulse duration of ≈30 fs and per‐pulse energy of ≈1–3 mJ. All data collection was performed in vacuum conditions and under room temperature. At the high photon energy of this experiment, the detection efficiency of the downstream spectrometer was low, and the spectrometer background caused significant errors in the measured photon energy. Thus, we implemented a new procedure for background (“pedestal”) subtraction for the downstream spectrometer. We averaged several thousand dark shots on the spectrometer and subtracted the result from all subsequent spectra.

## Conflict of Interest

The authors declare no conflict of interest.

## Supporting information

Supplementary Material

## Data Availability

The data that support the findings of this study are available from the corresponding author upon reasonable request.
