# Supplementary material for: Engineering Supramolecular Hybrid Architectures with Directional Organofluorine Bonds
Source: Small Sci. 2023 Dec 13;4(1):2300110. doi: 10.1002/smsc.202300110 (PMC11784642; doi:10.1002/smsc.202300110)
Supplement: Supplementary file 1 — Supplementary Material [file SMSC-4-2300110-s001.pdf]

Supporting Information for:

Engineering Supramolecular Hybrid Architectures with Directional Organofluorine Bonds

Patience A. Kotei,[1,2] Daniel W. Paley,[3] Vanessa Oklejas,[3] David W. Mittan-Moreau,[3] Elyse A. Schriber[1,2], Mariya Aleksich,[1,2] Maggie C. Willson,[1,2] Ichiro Inoue,[6] Shigeki Owada,[6,7] Kensuke Tono,[6,7] Michihiro Sugahara,[6] Satomi Inaba-Inoue,[7,8] Andrew Aquila,[4] Frédéric Poitevin,[4] Johannes P. Blaschke,[5] Stella Lisova,[4] Mark S. Hunter,[4] Raymond G. Sierra,[4] José A. Gascón,[2] Nicholas K. Sauter,[3] Aaron S. Brewster,*[3] J. Nathan Hohman*[1,2]

Author Affiliations:

[1]Institute of Materials Science, University of Connecticut, CT, 06269, USA

[2]Department of Chemistry, University of Connecticut, CT, 06269, USA

[3]Molecular Biophysics and Integrated Bioimaging Division, Lawrence Berkeley National Laboratory, Berkeley, CA, 94720, USA

[4]Linac Coherent Light Source, SLAC National Accelerator Laboratory, Menlo Park, CA, 94025, USA

[5]National Energy Research Scientific Computing Center, Lawrence Berkeley National Laboratory, Berkeley, CA, 94720, USA

[6]RIKEN SPring-8 Center, 1-1-1 Kouto, Sayo, Hyogo 679-5148, Japan

[7]Japan Synchrotron Radiation Research Institute, 1-1-1 Kouto, Sayo, Hyogo 679-5198, Japan

[8]Structural Biology Research Center, Photon Factory, Institute of Materials Structure Science, High Energy Accelerator Research Organization, 1-1 Oho, Tsukuba, Ibaraki, 305-0801, Japan

Table of Contents

1. **The 2F Polymorph: 2FY** 2
2. **Comment on Band gap analysis** 4
3. **TGA data** 4
4. **Density Functional Theory** 4
5. **Data Processing** 5

The 2F Polymorh: 2FY

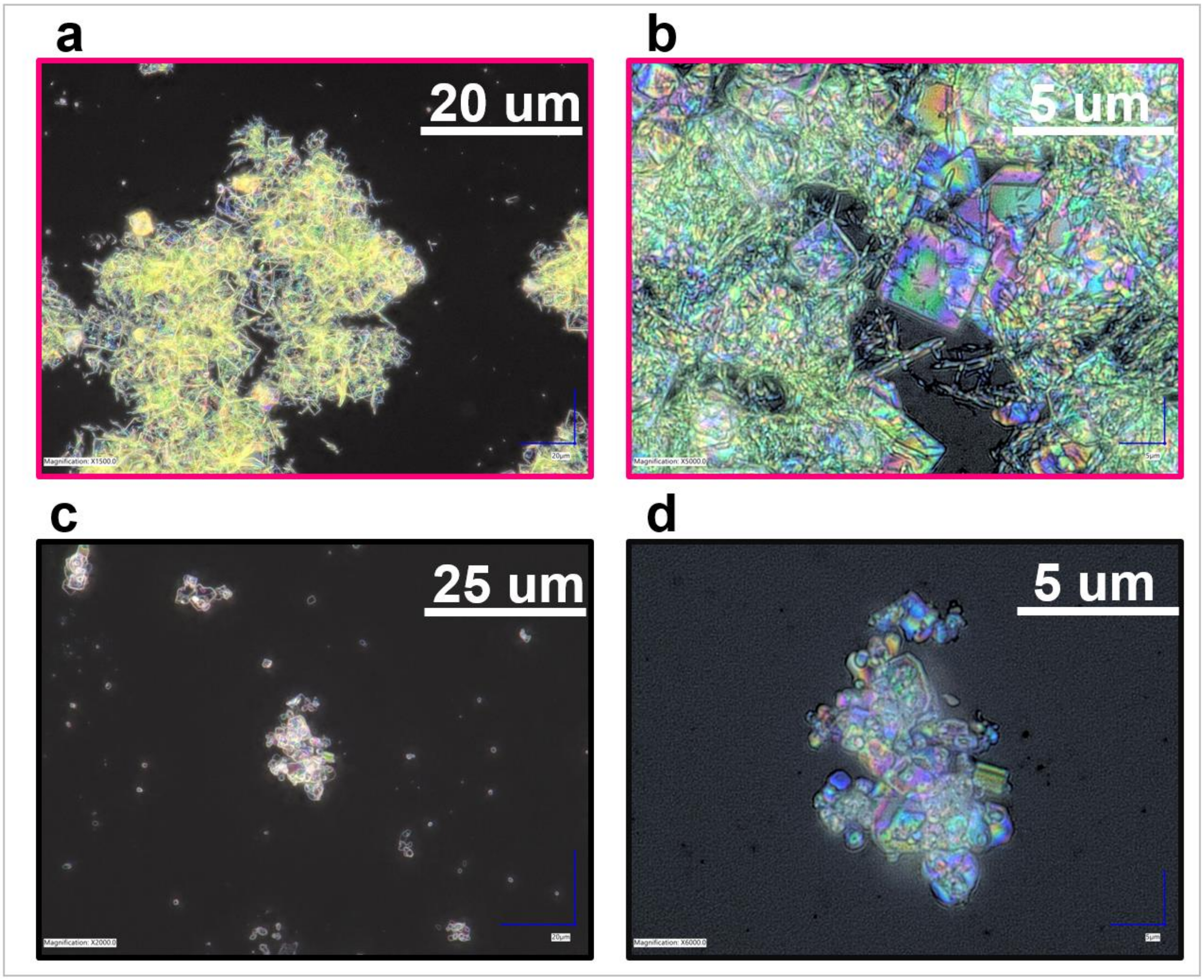

Figure s1| Optical variation in 2-FLUORO (a) and (b) a mixture of needlelike and tabular crystals observed in the 2FY product (c) and (d) colour and morphological change upon sonicating or heating the 2FY product after synthesis: a pure 2F phase is realized

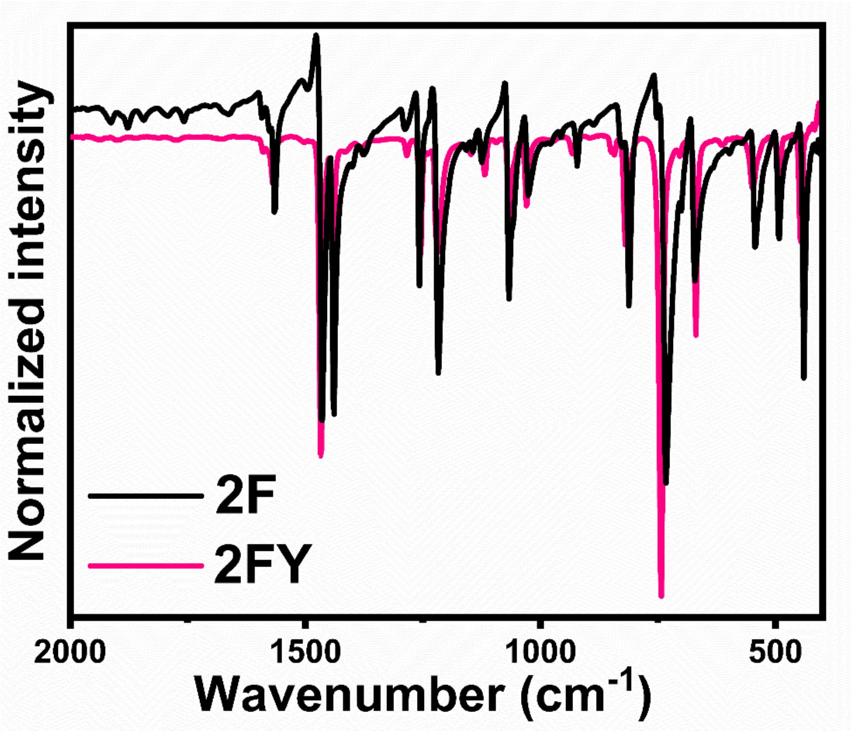

Figure s2| Infrared Spectra: Similar IR fingerprint observed in the two polymorphs with slight shift in peak positions

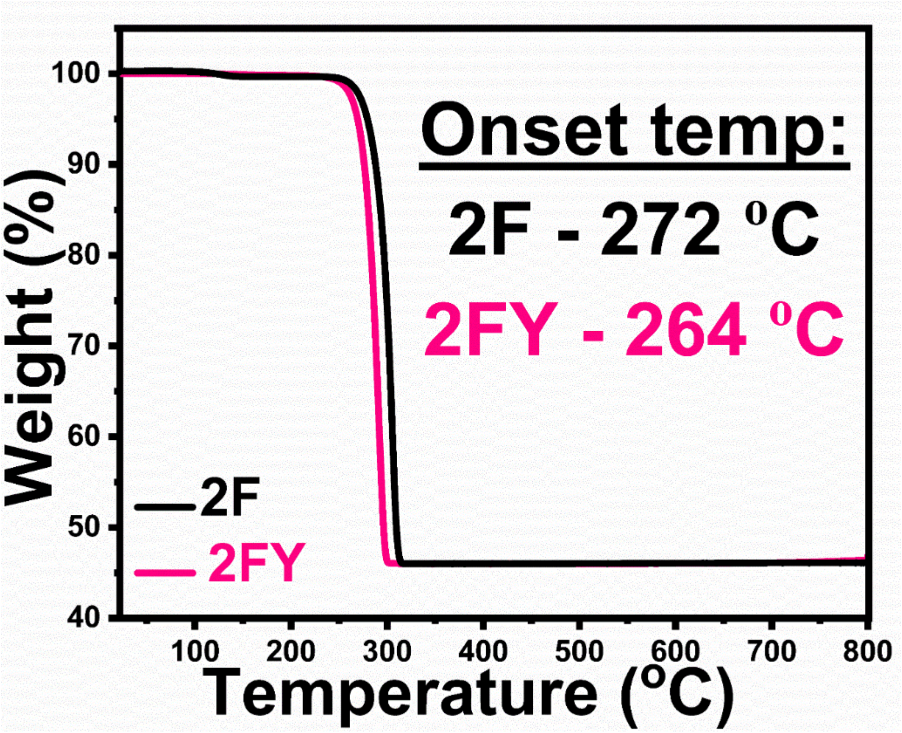

Figure s3| Thermal stability: a lower onset decomposition temperature is observed in 2FY compared the 2F product

Comment on Bandgap Analysis

Reflectance mode was used during diffuse reflectance spectroscopy analysis because the crystals were opaque solids, each of which shows a band absorption edge at a similar region. There was slight variance in band absorption edge of TH, 2F, 3F and 4F, resulting in a range of measured optical gaps of 3.0 and 3.2 eV, as shown in the insets of figure 2d. This variation may be evident of the tunable electronic band structure of this material class, that is, an inherent behavior of small adjustable bandgap ranges. Of the three compounds, TH (the archetype) has a weak feature in the reflectance mode measurements below the band edge that appears absent in the Fluoro-derivatized examples. Thus, we consider these compounds to be largely optically inactive.

Weight change and experimental residue obtained from the Thermogravimetric Analysis performed.

Table s1| experimental weight change and residue percentage obtained from thermal decomposition analysis.

**Weight change (%)**			
**TH**	**2F**	**3F**	**4F**
**50.00**	**54.26**	**52.80**	**54.00**
**Theoretical Residue - M.W(Ag)**		**45.90 %**	
**Experimental Residue (%)**			
**TH**	**2F**	**3F**	**4F**
**50.50**	**45.86**	**46.81**	**45.94**

Density Functional Theory

Table s2| DFT Geometric parameters and energies. Values in parenthesis correspond to the experimental crystallographic parameters. Both the C-F···H and Ag-Ag distances correspond to the shortest of each kind. The last column is the unit cell energy per atom, and it is referenced with respect to the value for **3F**.

	a **(Å)**	***b*** **(Å)**	***c*** **(Å)**	**C-F···H bond (Å)**	**Ag···Ag distance (Å)**	**Unit cell energy (a.u.)**	**Energy/ atom (kcal/mol)**
**2F**	6.751 (6.717)	6.751 (6.717)	27.569 (27.443)	2.55 (2.68)	3.38 (3.36)	-3535.863129	0.467
**3F**	7.570 (7.371)	5.686 (5.935)	14.844 (14.887)	2.25 (2.43)	2.92 (2.99)	-1767.970231	0.000
**4F**	5.547 (6.023)	7.532 (7.262)	30.005 (29.520)	2.46 (2.66)	3.01 (3.01)	-3535.909813	0.186

Data Processing

The structure determination follows steps that have been described previously[1,2]. Briefly, the following steps are required:

The detector internal metrology (relative positions of panels) was refined against a separately collected macromolecular dataset using the methods described in Brewster 2018. This step is necessary because the sparse diffraction patterns in smSFX datasets do not yield enough spot coverage to independently refine the positions of individual panels.

The Dials spotfinder was used to harvest diffraction spots from a subset of the full data. Typically, 5-10 minutes of data collection is enough for this step. The harvested spots are converted to d-spacings and synthesized into a virtual powder diffraction pattern as described previously. For LPD data collection, these runs were collected at a longer detector distance to improve the sharpness of the powder pattern. For Jungfrau 4M data, as a consequence of the smaller pixels and higher X-ray energy, a single detector position with inscribed resolution ~0.8 Å was sufficient for both unit cell determination and structure solution.

When synthesizing virtual powder patterns, we observed that small shifts in the detector position were common over the course of the experiment. This was especially notable at EuXFEL, where the detector position was readjusted manually after every distance change. The quality of powder patterns has a critical dependence on very accurate (~5-10 um) calibration of the beam center. Therefore, the beam center was adjusted as an integral part of the powder pattern creation. We identified a single isolated peak in the diffraction pattern and computed its width as a standard deviation of all individual observations of that peak. We then performed a grid search in decreasing steps (typically from 2 pixels to 1/32 pixel) until the peak sharpness was optimized.

After a properly calibrated virtual powder diffraction pattern was prepared, we manually selected 20-25 d-spacings from the pattern to use for unit cell determination. We obtained the most consistent results using the commercial crystallography program TOPAS-Academic. Often this resulted in several reasonable unit cell proposals. For the closely related series of unit cells described here, it was trivial to identify the matching cell, but for a fully unknown sample it is often helpful to attempt cctbx.small_cell indexing of a subset of the data with several unit cell candidates. The cell with the highest indexing rate is typically the correct one.

After TOPAS unit cell determination, we proceeded with cctbx.small_cell indexing, and integration as previously described. In the cctbx.xfel.merge scaling and merging step, we implemented a new weighting scheme for scaling individual frames, where the uncertainties on individual observations are assigned as 1/(Icalc^2 + sigma(I)^2) with sigma(I) representing the uncertainty on the measured net counts in a diffraction peak. This new weighting scheme is activated by setting scaling.weights=icalc_sigma as a parameter for cctbx.xfel.merge.

After merging, the ~0.8 Å resolution datasets were solved using ShelXT with the default settings. Refinement proceeded routinely using ShelXL. We refined an extinction parameter for all structures (ShelXL instruction EXTI), which is an empirical model for attenuation of the strongest structure factors. While we do not believe that literal crystallographic extinction is possible in serial microcrystal diffraction, the refinements with extinction resulted in more interpretable difference maps, so we argue that this technique is beneficial. At the end of the refinement process, we repeated the scaling and merging step with the working model (with isotropic displacement parameters) as a scaling reference as described previously. In the final refinements, hydrogen atoms were placed in calculated positions with riding isotropic displacement parameters; all other atoms were refined with unrestrained anisotropic displacement parameters.

References

[1] E. A. Schriber, D. W. Paley, R. Bolotovsky, D. J. Rosenberg, R. G. Sierra, A. Aquila, D. Mendez, F. Poitevin, J. P. Blaschke, A. Bhowmick, R. P. Kelly, M. Hunter, B. Hayes, D. C. Popple, M. Yeung, C. Pareja-Rivera, S. Lisova, K. Tono, M. Sugahara, S. Owada, T. Kuykendall, K. Yao, P. J. Schuck, D. Solis-Ibarra, N. K. Sauter, A. S. Brewster, J. N. Hohman*, Nature (London)* **2022***, 601*,7893, 360.

[2] M. Aleksich, D. W. Paley, E. A. Schriber, W. Linthicum, V. Oklejas, D. W. Mittan-Moreau, R. P. Kelly, P. A. Kotei, A. Ghodsi, R. G. Sierra, A. Aquila, F. Poitevin, J. P. Blaschke, M. Vakili, C. J. Milne, F. Dall'Antonia, D. Khakhulin, F. Ardana-Lamas, F. Lima, J. Valerio, H. Han, T. Gallo, H. Yousef, O. Turkot, I. J. Bermudez Macias, T. Kluyver, P. Schmidt, L. Gelisio, A. R. Round, Y. Jiang, D. Vinci, Y. Uemura, M. Kloos, M. Hunter, A. P. Mancuso, B. D. Huey, L. R. Parent, N. K. Sauter, A. S. Brewster, J. N. Hohman*, J. Am. Chem. Soc.* **2023***, 145*,31, 17042.
